# Transcriptome profiling of a novel *Enterobacter aerogenes* mutant with mannose-rich exopolysaccharide phenotype induced by phosphoenolpyruvate carboxylase inactivation

**DOI:** 10.1128/aem.01895-25

**Published:** 2026-03-19

**Authors:** Xinru Peng, Jiale Chen, Yilin Ding, Ruoxuan Bai, Ping Lu, Fangxu Xu, Guohong Zeng, Hongxin Zhao

**Affiliations:** 1 Zhejiang Province Key Laboratory of Plant Secondary Metabolism and Regulation, College of Life Sciences and Medicine, Zhejiang Sci-Tech University 12646, Hangzhou, China; 2 College of Life Science, Shenyang Normal University 12402, Shenyang, China; Kyoto University, Kyoto, Japan

**Keywords:** mannose, *Enterobacter aerogenes*, phosphoenolpyruvate carboxylase, polysaccharide, transcriptomic analysis

## Abstract

Mannose, a common monosaccharide with primary functions in protein glycosylation and immune regulation, has been widely used in medicine, cosmetics, and food additives, increasing its economic value. In our study, the *ppc* gene was knocked out in *Enterobacter aerogenes* IAM1183 to regulate phosphoenolpyruvate carboxylase activity, thereby optimizing hydrogen-producing metabolism. Unexpectedly, the mutant strain exhibited successful production of a substantial amount of polysaccharides with a yield of 6.75 ± 0.20 g∙L^−1^, a feature not observed in the original strain. Notably, the four main monosaccharides of the detected polysaccharide were mannose, glucuronic acid, glucose, and galactose, with proportions of 50.28%, 24.15%, 18.35%, and 7.2%, respectively. According to the transcriptional analysis, 481 genes were significantly differentially expressed, 47% of which were metabolism-related, encoding key enzymes of amino acid and carbohydrate metabolism, phosphotransferase system, and exopolysaccharide synthesis. It was speculated that the deletion of *ppc* not only enhanced the extracellular polysaccharide synthesis pathway but also affected the transport and phosphorylation of carbohydrates in cells, resulting in a distinct polysaccharide production phenotype. In this study, the production of mannose-rich exopolysaccharide (EPS) in *E. aerogenes* was realized for the first time. The high-mannose content of this EPS endows the mutant strain with significant potential as a candidate for downstream mannose extraction and utilization. Concurrently, this work delineates a pathway to mannose-rich EPS production in *E. aerogenes*, thereby enhancing the understanding of its metabolic network in this non-model microorganism.

**IMPORTANCE:**

Mannose is a crucial monosaccharide with diverse applications in multiple industries, yet current production methods have limitations. Our study is of great importance as it represents the first instance of mannose-rich polysaccharides being identified in *Enterobacter aerogenes* when hydrogen production metabolism is optimized by knocking out the *ppc* gene. Transcriptomic analysis suggested that the *ppc* gene knockout affected numerous genes related to metabolism, which is crucial for further exploring the metabolic regulation mechanism of *Enterobacter aerogenes*. This study reports a novel genetically engineered strain and a systematic methodology for mannose biosynthesis, thereby identifying candidate regulatory nodes within its metabolic network in this non-model microorganism.

## INTRODUCTION

Mannose is a hexose that often occurs in nature in the form of mannan polysaccharides. In recent years, mannose has been widely used in food (1), medicine (2), cosmetics (3), as well as other fields (4, 5), and the economic development value of mannose production methods has become increasingly prominent. Mannose is often used as a low-calorie sweetener in foods and beverages (6). In biomedicine, mannose not only has the effect of regulating cellular immunity (7), protecting against pathogen infection (8), and promoting glycoprotein synthesis (9) but is also used to synthesize pharmaceutical intermediates such as carbohydrate drug precursors (10).

At present, the main methods for preparing mannose are plant extraction, chemical synthesis, and biological fermentation (11). Tang et al. (12) developed a mannose preparation process using dragon fruit stem as raw material via a series of processing steps, such as ultrahigh pressure-assisted hot water extraction and enzymatic hydrolysis. The purity of the resulting mannose preparation can reach more than 99%, but the process route is complicated, and the yield is low. Fang et al. (13) produced mannose from palm fruit residue by microwave-assisted extraction combined with sulfuric acid treatment. The mannose yield reached up to 92%, but this method required a large amount of organic reagents, making it environmentally unfriendly. Alternatively, mannose can also be chemically produced from glucose. Han et al. (14) utilized a metal-organic framework (MOF) incorporating Keggin-type phosphomolybdate to catalyze the isomerization of glucose. Under optimal conditions, an excellent mannose yield of up to 32.5% with an impressive selectivity of 94% was achieved. In addition, by combining ammonium molybdate and calcium oxide as a catalyst, the mannose yield was increased to 44.8% after reaction at 150°C and pH 3.0 for 80 min (15). Compared with plant extraction, chemical synthesis is simpler and more convenient, but it requires harsh reaction conditions, while also generating large numbers of by-products, causing separation difficulties and increasing the cost of downstream processing. However, both extraction and chemical synthesis approaches are limited in scalability and sustainability, as they rely on either agricultural residues or energy-intensive catalytic processes (15–17). In contrast, biological fermentation offers a greener and potentially scalable route under mild conditions, with lower energy input and fewer environmental pollutants, although its current efficiency and yields remain to be optimized (1, 11).

In recent years, the production of chemicals using biological fermentation methods has been widely investigated by researchers, with several studies focusing on mannose or mannose-rich polysaccharides. For example, Palur et al. constructed a system to generate D-mannose through the phosphorylation-isomerization-dephosphorylation pathway in *E. coli*, with a conversion rate of the total sugar in the product of approximately 52.1%. The yield of the D-mannose fraction was approximately 21.3% (18). Moreover, a number of Lactobacillus strains have been used for the production of mannose-type exopolysaccharides (19, 20). Nevertheless, only a few studies have so far explored biological routes to D-mannose, and the available quantitative data on fermentation titers and yields remain limited (11, 17).

*E. aerogenes* is an anaerobic gram-negative bacterium, which has the advantages of fast growth, wide substrate spectrum, high-density culture, etc., and is considered one of the most promising strains for industrial applications (21). Currently, *E. aerogenes* is mainly used in biological fermentation to produce hydrogen (22) and 2, 3-butanediol (23), while the production of mannose using this strain has not been reported.

The *ppc* gene encodes phosphoenolpyruvate carboxylase (PEPC), one of the most important enzymes in central metabolism (24). It is a cytoplasmic enzyme that converts phosphoenolpyruvate (PEP) and HCO_3_^−^ into oxaloacetate (25), which enters the tricarboxylic acid cycle (26). PEPC is also involved in the regulation of carbon flux from carbohydrates toward fatty acids and proteins (27, 28).

In this study, *E. aerogenes* IAM1183 (Ea), a highly efficient hydrogen-producing strain (29), was genetically modified through triparental conjugation to disrupt the *ppc* gene, thereby blocking the conversion of phosphoenolpyruvate (PEP) into oxaloacetate. This genetic alteration was intended to redirect metabolic flux by increasing the availability of PEP for pyruvate synthesis, which may subsequently enhance pyruvate conversion to formate via pyruvate formate-lyase (PFL). The generated formate was then expected to be decomposed into CO_2_ and H_2_ by formate hydrogen lyase (FHL), potentially leading to increased hydrogen production. Surprisingly, however, the mutant strain exhibited a pronounced exopolysaccharide (EPS) phenotype characterized by a mannose-dominant composition, a trait absent in the parental strain.

To our best knowledge, this is the first report of mannose-dominant EPS production in this organism. The study is not to produce free mannose *per se*, but to characterize this mannose-rich EPS phenotype, decode the transcriptional regulatory networks, and validate *E. aerogenes* as a promising strain for functional sugar production. Although mannose has not been isolated and purified from this extracellular polysaccharide, the high mannose content (50.28%) of this extracellular polysaccharide makes this mutant a potential candidate strain for subsequent mannose extraction and utilization. Altogether, this work identifies a route to mannose-rich EPS in *E. aerogenes*, contributing to the understanding of the metabolic network of this non-model microorganism, and provides a foundation for downstream recovery of mannose-containing products.

## MATERIALS AND METHODS

### Strains and plasmids

The strains and plasmids used in this study are listed in Table 1. All engineered strains were derived from wild-type *E. aerogenes* IAM1183 (21). *E. coli* DH5α and BL21 were used for cloning and protein expression, respectively. The recombinant plasmids were purchased from Vazyme Biotechnology Co., LTD., Nanjing, China.

**TABLE 1:** Strains and plasmids used in this study

Strain or plasmid	Genotype and relevant characteristics	Source or reference
Strains		
*E. coli* S17-1 λpir	RP4-2 (Km::Tn7,Tc::Mu-1), *pro*-82, *LAMpir*, *recA*1, *endA*1, *thiE*1, *hsdR*17, *creC*510; mobilizer strain	Beyotime
*E. aerogenes* IAM1183	Wild type	IAM (Tokyo, Japan)
*E. coli* HB101	Carrying pRK415; Km^r^	(1)
*E. coli* DH5α	F^-^Φ80d/*lac*ZΔM15, Δ(*lac*ZYA-*arg*F) U169, *recA*1, *endA*1, *hsdR*17 (r_K_^–^, m_K_^+^), *phoA*, *supE*44, λ-, *thi*-1, *gyrA*96, *relA*1	Vazyme
*E. coli* BL21	F^–^, *omp*T, *hsd*S_B_ (r_B_^−^ m_B_^−^), *gal*, *dcm* (DE3)	Vazyme
EaΔppc	*E. aerogenes* IAM1183Δ*ppc*, Amp^r^	This study
EaΔppc/p	*E. aerogenes* IAM1183Δ*ppc* carrying plasmid pET-28a-ppc, Amp^r^	This study
Plasmids		
pLO3-ppc	pLO3 carrying upstream 664 bp and downstream 578 bp of *ppc*	This study
pET-28a	Kan^r^	This lab
pKD46-cm	Chl^r^*,* derived from pKD46	This lab
pCP20-cm	Chl^r^*,* derived from pCP20	This lab
pKD4	Kan^r^*,* FRT site	This lab
pET-28a-ppc	Kan^r^, derived from pET-28a	This study

### Media and culture conditions

Luria-Bertani (LB) medium (10 g·L^−1^ tryptone, 5 g·L^−1^ yeast extract, 10 g·L^−1^ NaCl) was used for routine culture. For strains carrying selection markers, ampicillin (100 mg·L^−1^), kanamycin (50 mg·L^−1^), or chloramphenicol (25 mg·L^−1^) was added where appropriate.

Yeast malt (YM) solid medium contained 5 g·L^−1^ peptone, 3 g·L^−1^ yeast extract, 3 g·L^−1^ malt extract, 10 g·L^−1^ glucose, and 18·g L^−1^ agar. It was autoclaved at 115°C for 30 min and cooled to 45°C, after which ampicillin (100 mg·L^−1^), streptomycin sulfate (25 mg·L^−1^), and tetracycline (25 mg·L^−1^) were added before pouring into plates.

The 8% sucrose solid medium contained 5 g·L^−1^ peptone, 2 g·L^−1^ yeast extract, 3 g·L^−1^ beef extract, 1 g·L^−1^ NaCl, 80 g·L^−1^ sucrose, and 18 g·L^−1^ agar. It was autoclaved at 121°C for 20 min and poured into plates.

Aerobic fermentation medium with glucose as carbon source contained 30 g·L^−1^ glucose, 3 g·L^−1^ KH_2_PO_4_, 6.8 g·L^−1^ Na_2_HPO_4_, 0.75 g·L^−1^ KCl, 5.35 g·L^−1^ (NH_4_)_2_SO_4_, 0.28 g·L^−1^ Na_2_SO_4_, 0.26 g·L^−1^ MgSO_4_·7H_2_O, 0.42 g·L^−1^ citric acid, 5 g·L^−1^ yeast extract, and 0.3 mL·L^−1^ trace element solution (34.2 g·L^−1^ ZnCl_2_, 2.7 g·L^−1^ FeCl_3_·6H_2_O, 10 g·L^−1^ MnCl_4_·4H_2_O, 0.85 g·L^−1^ CuSO_4_·H_2_O, and 0.31 g·L^−1^ H_3_BO_3_). Unless indicated otherwise, all reagents were purchased from Sigma-Aldrich (St. Louis, MO, USA).

### Construction of gene deletion mutants

Genomic DNA of Ea was extracted using the Ezup column bacterial genomic DNA extraction kit (Sangon Biotech, Shanghai, China). The up- and downstream flanking sequences of the *ppc* gene were amplified by PCR using the genomic DNA as the template with the ppc-UF/UR and ppc-DF/DR primer pairs (Table 2), respectively, and the fragments were purified using a SanPrep column DNA gel recovery kit (Sangon Biotech, Shanghai, China). Using these two fragments of DNA as the template with ppc-UF and ppc-DR as primers (Table 2), overlap-extension PCR was used to amplify a cassette without the *ppc* gene, which was inserted into the pLO3 plasmid using *Sac*I/*Xba*I restriction endonucleases and T4 ligase (Takara, Beijing, China).

**TABLE 2:** Primers selected and used in this study^*a*^

Primer	Sequence (5′ to 3′)
ppc-UF	TAGGAGCTCACGCCTCAAACCGCATCTGC (*Sac*I)
ppc-UR	TACCGGGAACGGCTGATAAAGTTACCCCAGACACCCCATC
ppc-DF	GATGGGGTGTCTGGGGTAACTTTATCAGCCGTTCCCGGTA
ppc-DR	TGCTCTAGAGGATCTGGCAGTCGGTGA (*Xba*I)
SacB-VF	AGCGAAGTGTGAGTAAGTAAAG
SacB-VR	CGAACCAAAAGCCATATAAG
ppcpet-F	CCGGAATTCATGAACGAACAATATTCCGC (*EcoR*I)
ppcpet-R	CCCAAGCTTTTAGCCCGTGTTGCGCATAC (*Hind*III)
QrecA-F	ATTCTTTACGGCGAAGGTATCAA
QrecA-R	CGGGTTATCTTTCAGCCACGA
Qppc-F	AGTATGCTCGGTAAGGTGCTAGG
Qppc-R	TTCCTGGCGATTGGCTTCATTG

^*a*^ Relevant restriction enzyme sites are underlined.

The resulting recombinant plasmid was introduced into *E. coli* S17-1, which served as the conjugational donor strain. Cells of S17-1/pLO3-ppc, HB101/pRK415, and Ea were washed and re-suspended, mixed in equal volume, and then captured on a 0.45 μm sterile filter membrane, which was placed on LB solid medium and cultured at 37°C for 12 h. After washing the filter membrane and re-suspending, the bacterial suspension was spread onto a YM agar plate and cultured at 37°C for 12 h. The clones were analyzed for the presence of the *sacB* gene using the SacB-VF/VR primer pair (Table 2) to verify whether the pLO3-ppc vector was successfully transferred into Ea. The transconjugants were picked and grown in LB medium at 37°C and 220 rpm. After three rounds of passaging, the bacterial suspension was subjected to gradient-dilution up to 10^−7^ and cultured on 8% sucrose plates at 37°C for 12 h to screen recombinant strains with double homologous recombination exchanges. Only the strains lacking the *sacB* gene, which encodes levansucrase, could grow on a sucrose plate, serving as a screening marker for the second homologous recombination. The final recombinants were verified by colony PCR and Sanger sequencing (Sangon Biotech, Shanghai, China) using the validation primers ppc-UF/DR (Table 2).

### Construction of the *ppc* gene complementation strain

To verify the effect of *ppc* knockout on the production of polysaccharides, the recombinant plasmid pET28a-ppc was constructed to restore the expression of PEPC. The total RNA of Ea, EaΔppc, and EaΔppc/p strains was extracted using the Trizol reagent (Thermo Fisher Scientific, USA). After electrophoretic verification, reverse transcription was performed using Rever Tra Ace qPCR RT Master Mix (Toyobo, Japan). The *recA* gene was selected as an internal reference for RT-qPCR to measure the expression levels of the *ppc* gene in the three strains.

### Fermentation of polysaccharides

The recombinant strain EaΔppc was grown in a 250 mL shake flask containing 50 mL of LB liquid medium at 37°C and 220 rpm for 12 h. The resulting seed culture was used to inoculate aerobic fermentation medium, followed by incubation at 37°C and 220 rpm for 12 h. Then, the fermentation broth was centrifuged at 4°C and 8,000 × *g* for 30 min to remove the bacterial pellet, while the supernatant was stirred in a threefold volume of ethanol at 4°C and left overnight to obtain a white viscous precipitate. The precipitate was harvested by centrifugation and redissolved in ddH_2_O, after which proteins were removed by the Sevag method. After a second round of ethanol precipitation overnight, purified exopolysaccharides were obtained by centrifugation and dried by lyophilization (−40°C for 48 h).

### Detection of fermentation broth viscosity

The recombinant strain EaΔppc was grown in a 250 mL shake flask containing 50 mL of LB liquid medium at 37°C and 220 rpm for 12 h. The resulting seed culture was used to inoculate aerobic fermentation medium, followed by incubation at 37°C and 220 rpm for 12 h. Then, the fermentation broth was centrifuged at 4°C and 8,000 × *g* for 30 min to remove the bacterial pellet. The viscosity of the supernatant was measured using a Fungilab Viscolead OneL 100013 (Julabo-Visco Analysis and Testing Technology, New York, USA) equipped with an L4 spindle at a rotational speed of 100 rpm.

### Determination of residual glucose

The supernatant after ethanol precipitation of the polysaccharides was used to detect the residual glucose. Ethanol was removed at 50°C by using a rotary evaporator RE-52AA (Biochemical Instrument Factory of Shanghai Yarong, China) to avoid reacting with the DNS (3,5-Dinitrosalicylic Acid, Solarbio, Beijing) reagent and thereby affecting the detection of residual glucose concentration. 1 mL sample mixing with 2 mL DNS reagent was subjected to a boiling water bath for 5 min, then rapidly cooled to room temperature with cold water, and made up to 10 mL with distilled water. The absorbance was detected by a UV756CRT spectrophotometer (Beijing, China) at a wavelength of 540 nm.

### Polysaccharide molecular weight determination

The calibration curve of polysaccharide molecular weights was prepared using dextran standards with different molecular weights. The standard was prepared as a 5 mg∙mL^−1^ standard solution in 0.05 M NaCl solution and passed through a 0.22 μm pore-size filter membrane. The standards and samples were subjected to high-performance gel permeation chromatography (HPGPC), and the results were analyzed by Waters Empower software. OHpak SB-803 HQ, Ohpak SB-804 HQ, and Ohpak SB-805 HQ (8 × 300 mm) columns (AB Sciex, USA) were used in series with a differential detector and water-soluble SEC columns on the polymer matrix. The mobile phase was 0.05 M NaCl at a flow rate of 0.6 mL∙min^−1^, the column temperature was 40°C, and the sample size was 30 μL. The gel permeation chromatography and analysis were performed by Wuhan Huaster (Wuhan, China).

### Determination of carbohydrate composition by HPLC

Interestingly, the anticipated significant increase in hydrogen production was not observed in the mutant strain. Instead, the mutated strain displayed a viscous, gel-like phenotypic trait. To verify whether this phenotype was directly attributable to the deletion of the *ppc* gene, a gene complementation experiment was conducted for validation. Standard solutions of 10 types of monosaccharides, including fucose, rhamnose, arabinose, galactose, glucose, xylose, mannose, ribose, galacturonic acid, and glucuronic acid, were prepared at a concentration of 5 ± 0.05 mg∙mL^−1^. The exopolysaccharide sample was dissolved in 1 mL of 2 M trifluoroacetic acid and heated at 121°C for 2 h. The resulting hydrolysate was washed with 3 mL of methanol three times, after which 5 mL of sterile water was added to dissolve the sample to be tested.

A centrifuge tube was filled with 0.2 mL of the standard or the sample solution to be tested, after which 0.2 mL of 0.5 M aqueous NaOH and 0.5 mL of 0.5 M 1-phenyl-3-methyl-5-pyrazolone (PMP) methanol solution were added, mixed by vortexing, and reacted in a water bath at 70°C for 1 h. After the reaction was completed, 0.2 mL of 0.5 M HCl was added to neutralize the solution, followed by the addition of 1 mL of chloroform and vortexing to extract the excess PMP. Finally, 0.3 mL of the sample solution was removed into a sample vial, which was filled with water to 1 mL.

HPLC was performed on a Thermo U3000 liquid chromatography system (Thermo Fisher Scientific, USA) equipped with a ZORBAX Eclipse XDB-C18 column. The mobile phase comprised acetonitrile/phosphate buffer pH 6.8 (1:1) at a flow rate of 0.8 mL∙min^−1^. The column temperature was 30°C, and the detection wavelength was 250 nm. The injection volume was 10 μL.

### Transcriptome analysis of the wild-type and mutant strain

RNA-seq of the Ea and EaΔppc strains was performed on an Illumina HiSeq platform. Total RNA was extracted using the Trizol method, and the concentration and purity of the extracted RNA were assessed using a NanoDrop 2000 (Thermo Fisher Scientific, USA). The integrity of RNA was assessed by agarose gel electrophoresis. Rever Tra Ace qPCR RT Master Mix (Toyobo, Japan) with gDNA Remover was used to reverse-transcribe the obtained high-quality RNA into cDNA. The data generated by the Illumina platform were used for gene expression analysis, differential expression analysis, Gene Ontology (GO) enrichment analysis, and Kyoto Encyclopedia of Genes and Genomes (KEGG) enrichment analysis. All bioinformatics analyses were carried out on the cloud platform of Majorbio (Shanghai, China). To further verify the reliability of the transcriptomic results, real-time fluorescence quantitative PCR (RT-qPCR) analysis was performed for differentially expressed genes (DEGs) in the mutant, using *act1* as the internal reference gene. SYBR Green Realtime PCR Master Mix (Toyobo, Japan) was used for RT-qPCR.

## RESULTS AND DISCUSSION

### Construction of the EaΔppc knockout strain

**Fig 1:**
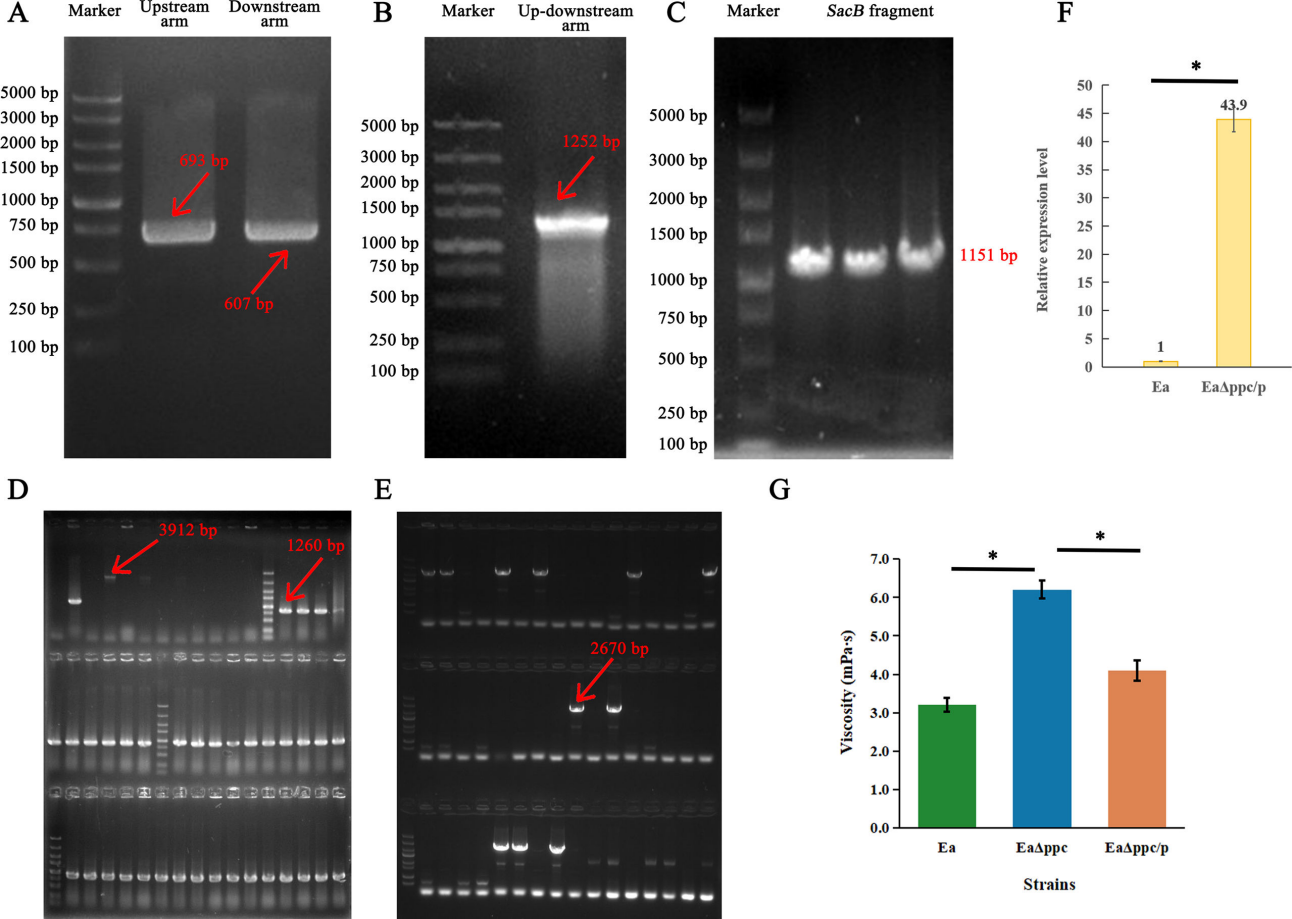
Construction of EaΔppc and EaΔppc/p. (**A**) Amplification of the *ppc* gene upstream and downstream arms. (**B**) Overlap-extension of the *ppc* gene upstream and downstream arm. (**C**) Amplification of the *sacB* gene verified whether the pLO3-ppc plasmids were connected to the genome by single homologous recombination. (**D**) 48 colonies were verified by amplification of the *ppc* gene upstream and downstream, positive recombinants showed specific bands in 1,260 bp, and false-positive recombinants showed faint bands in 3,912 bp (Marker: 5,000 bp, 3,000 bp, 2,000 bp, 1,500 bp, 1,000 bp, 750 bp, 500 bp, 250 bp, 100 bp). (**E**) 48 colonies were verified by amplification of the *ppc* gene, positive recombinants showed specific bands in 2,670 bp (Marker: 5,000 bp, 3,000 bp, 2,000 bp, 1,500 bp, 1,000 bp, 800 bp, 500 bp, 300 bp). (**F**) The relative expression level of *ppc* in the over-expression strain EaΔppc/p increased by 43 times compared with the original strain Ea. (**G**) Viscosity of Ea, EaΔppc, and the complemented strain EaΔppc/p. Data are shown as mean ± SD from three independent biological replicates (*n* = 3). Statistical significance among strains was assessed by one-way ANOVA followed by Tukey’s multiple-comparisons test. *P* < 0.05 was considered statistically significant (* indicates *P* < 0.05).

The positive transconjugant obtained after recombination was amplified to validate the target fragment, which was subsequently sequenced, and the result was consistent with the expected up- and downstream fragments of the *ppc* gene with the correct knockout site (Fig. 1A through D). The results, therefore, showed that the EaΔppc mutant was obtained by deleting the gene encoding phosphoenolpyruvate carboxylase (EC 4.1.1.31) in the genome of Ea.

### Gene complementation analysis

The open reading frame of the *ppc* gene was amplified from genome DNA using the primer pair ppcpet-F/R (Table 2), and the PCR fragment ligated into the plasmid pET28a linearized with *Eco*RI and *Hin*dIII (Fig. 1E). After plasmid induction, the total RNA of the EaΔppc/p strain was extracted and the expression level of the *ppc* gene was determined (Fig. 1F). Compared with the original strain, the relative expression level of *ppc* in the complementation strain was increased 43 times.

The subsequent culture of the strains showed that the colony morphology of EaΔppc/p on plates had changed, but there were still some sticky gel-like polysaccharides in the fermentation flask. These results indicated that the complementation weakened the production of polysaccharides to a certain extent, but the metabolic effects of gene knockout could not be fully compensated.

### Analysis of relative viscosity

The relative viscosity of Ea, EaΔppc, and EaΔppc/p was measured in the fermentation broth. The viscosities of the fermentation broth of the three strains were not very high in terms of values, but there were certain differences among them. Compared with the Ea, EaΔppc had a 93.75% increase in viscosity. After *ppc* gene complementation, the viscosity decreased by only 27.81%, higher than Ea (Fig. 1G). Differences among the three strains were evaluated using one-way ANOVA followed by Tukey’s multiple-comparisons test (*n* = 3 biological replicates; *P* < 0.05). This result indicated that the production of adhesive polysaccharides is indeed caused by the knockout of the *ppc* gene. While gene complementation can reduce the production of polysaccharides to a certain degree, it cannot completely compensate for the impact of *ppc* gene knockout on the metabolic pathways of Ea.

### Glucose consumption with polysaccharide production

Under aerobic fermentation, Ea did not produce any polysaccharides at all. In contrast, the *ppc* gene knockout strain produced viscous polysaccharides reaching 6.75 ± 0.20 g∙L^−1^, and the consumption of glucose increased significantly from 14.15 ± 0.35 g∙L^−1^ to 22.41 ± 0.62 g∙L^−1^ (Table 3). However, the increased glucose consumption was not fully utilized for polysaccharide synthesis. This might be due to the impact of *ppc* gene knockout on the TCA cycle and pyruvate metabolism, which aggravated the metabolic burden of the cell. These results suggested that the *ppc* knockout enhanced glucose utilization efficiency, though not all additional glucose was converted into polysaccharides.

**TABLE 3:** Molecular weight of polysaccharide

Sample name	Retention time	MP (Da)	Mw (Da)	Mn (Da)	Polymer dispersity index (PDI)	Proportion (%)	Mz	Mz + 1	Mz/Mw	Mz + 1/Mw
EaΔppc	44.928	1,148	1,258	1244	1.011	100	1,273	1,289	1.012	1.025

### Molecular weight of polysaccharides

**Fig 2:**
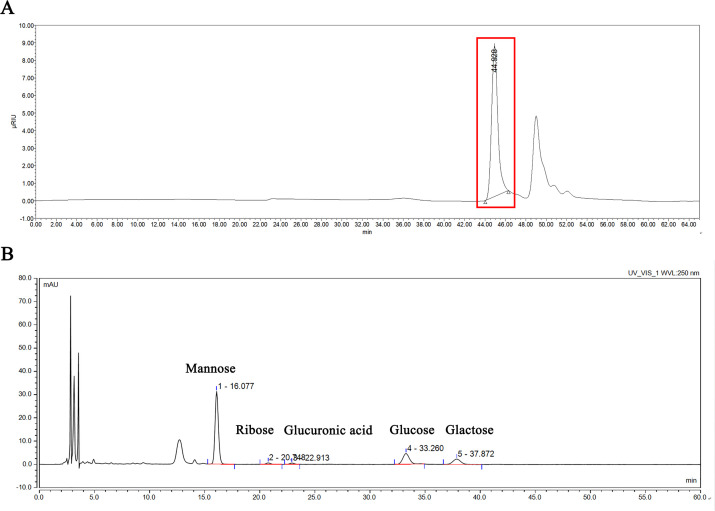
High-performance gel permeation chromatography and monosaccharide composition of polysaccharide. (**A**) A single symmetrical peak at 44.928 min, which indicated a homogeneous polysaccharide, and the solvent salt peak was at 49 min. (**B**) Four specific peaks indicated that the polysaccharide was composed of mannose, glucuronic acid, glucose, and galactose as its monosaccharide components (ribose was an impurity).

The results of HPGPC (Fig. 2A; Table 3) showed that the mutant strain produced a single kind of small polysaccharide, with a peak molecular weight (MP) of 1,148 Da, number average molecular weight (Mn) of 1,244 Da, weight average molecular weight (Mw) of 1,258 Da, and polydispersity index (PDI) of 1.011, indicating that the molecular weight distribution was uniform and the chemical purity was high.

### Identification of exopolysaccharides

The monosaccharide content of exopolysaccharides was quantitatively determined by HPLC using external standards. The composition of the exopolysaccharides is shown in Table 4. Four main monosaccharides were detected, among which mannose accounted for 50.3% (25.3 µg∙mg^−1^) of the total (Fig. 2B). As a control, the wild-type Ea strain was subjected to the same procedure to extract exopolysaccharides. After ethanol precipitation, no white viscous precipitate was obtained from the fermentation solution.

**TABLE 4:** Content of each component of polysaccharide

Total content (mg g^−1^)	Proportion of each component (%)									
	Fuc	Rha	Ara	Gal	Glc	Xyl	Man	Rib	Gal-UA	Glc-UA
50.383	0.000	0.000	0.000	18.357	24.155	0.000	50.288	0.000	0.000	7.200

### Analysis of principal component analysis and differentially expressed genes

**Fig 3:**
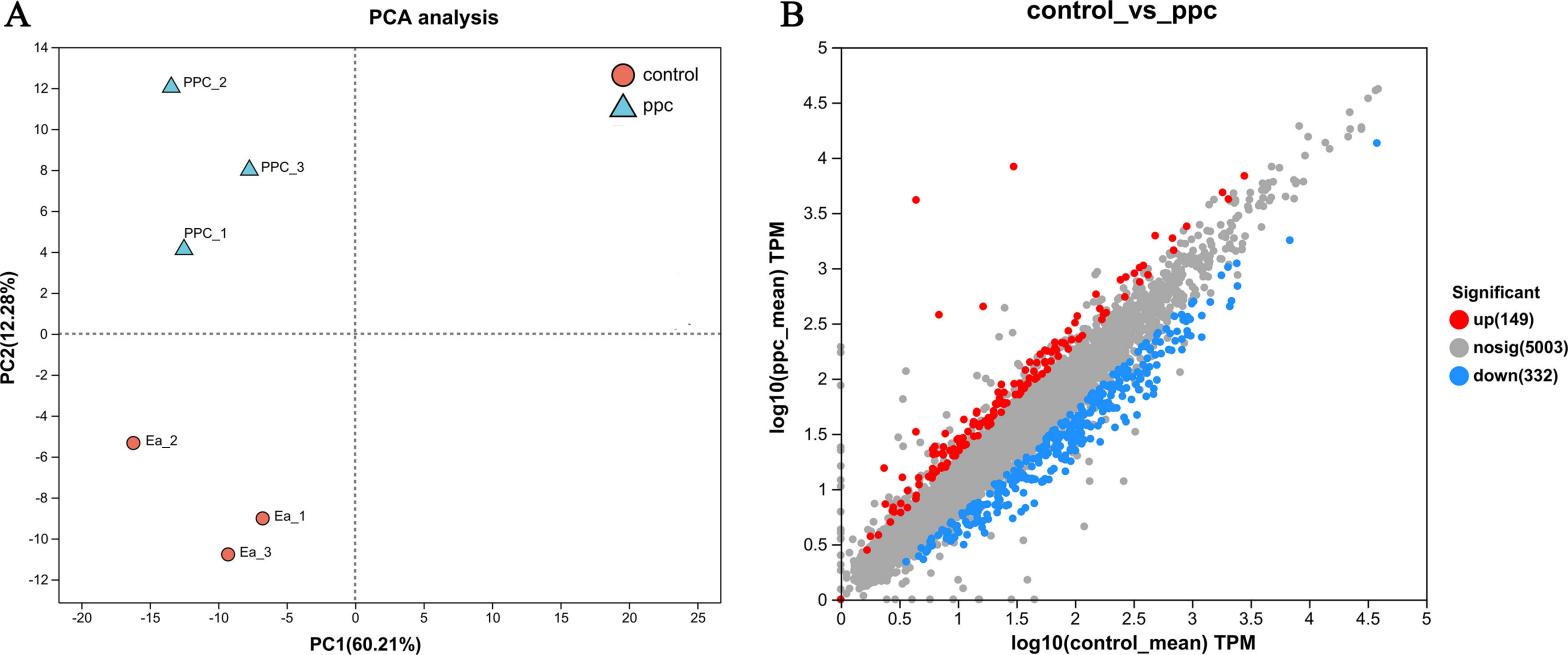
Preliminary analysis of gene expression between Ea and EaΔppc. (**A**) PCA showed that the expression of the Ea and EaΔppc was very different, and the triplicate of the same strain was repeatable. (**B**) Scatter plot of expression difference between Ea and EaΔppc, there were significant differences in the expression of 481 genes (FDR ≤ 0.05 and FC ≥ 2), among which 149 were up- and 332 downregulated.

The unexpected phenotypes observed after *ppc* gene knockout implied disruptions in the regulatory network’s gene expression patterns. To elucidate these alterations, transcriptome analysis was conducted to compare global gene expression profiles between *E. aerogenes* (Ea) and the *ppc*-deficient mutant strain EaΔppc, aiming to identify the transcriptional changes underpinning the observed phenotypic shifts. Principal component analysis (PCA) was used to assess the overall gene expression differences between the Ea and EaΔppc strains. The trend of aggregation and separation of samples was visually represented in the plot, where more aggregated points indicated greater similarity in the observed variables, and vice versa (Fig. 3A).

There were significant differences in the expression of 481 genes (FDR ≤ 0.05 and FC ≥ 2), among which 149 were up- and 332 downregulated. The detailed information on the DEGs is shown in the scatterplot in Fig. 3B.

### Analysis of GO annotation and KEGG pathways

**Fig 4:**
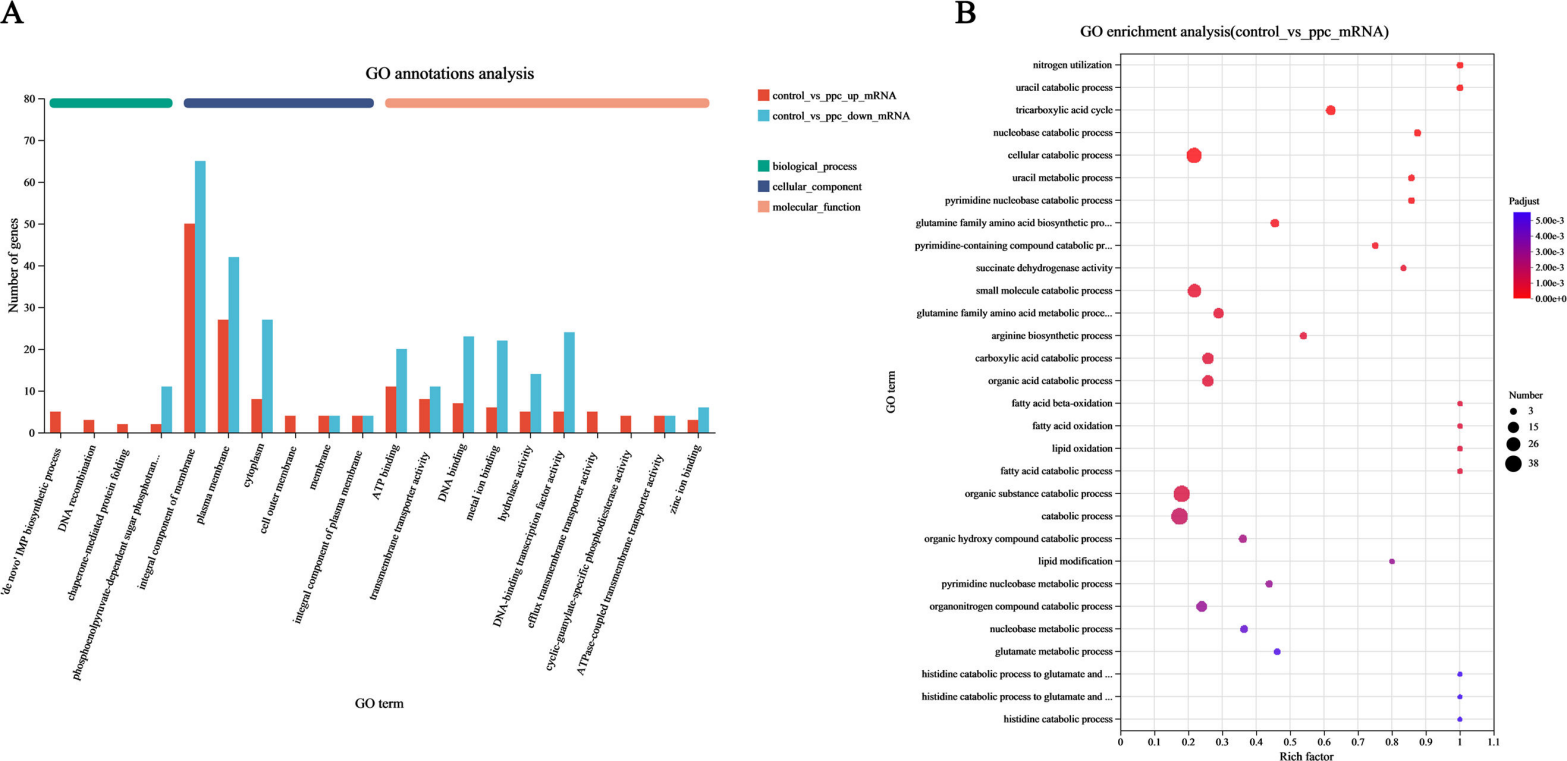
Ea and EaΔppc GO analysis of DEGs. (**A**) GO functional classification statistical histogram of DEGs was carried out. The genes with significant upregulation and downregulation changes were mainly those belonging to the cellular component. (**B**) The GO enrichment bubble diagram showed that multiple functions were enriched, indicating the differentiation between Ea and EaΔppc.

To further understand the changes in gene expression, functional annotation of DEGs was conducted using GO analysis. The results showed that genes with significant up- and downregulation were mainly associated with the integral components of the membrane belonging to the cellular component category, accounting for 23.9% of all DEGs (Fig. 4A). Among the metabolism-related DEGs, 65 were up- and 50 downregulated. Further GO enrichment analysis indicated that the DEGs were mainly related to catabolic processes (Fig. 4B).

**Fig 5:**
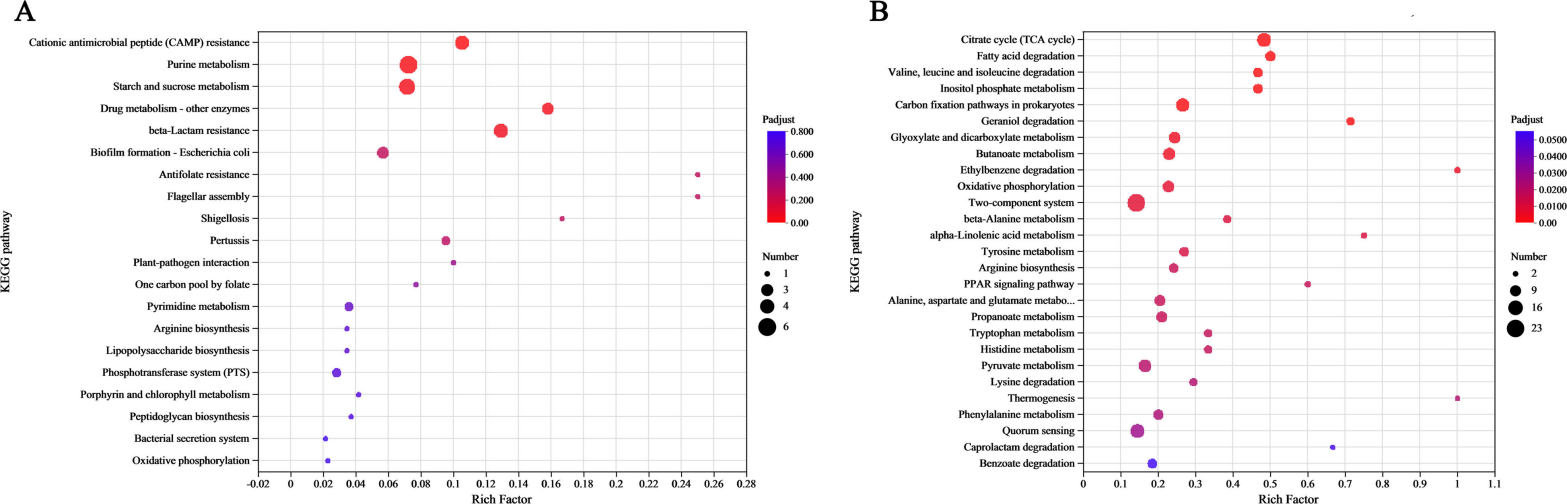
KEGG pathway enrichment analysis of DEGs in Ea and EaΔppc. (**A**) Upregulated DEGs were significantly enriched in purine metabolism (six genes) and starch and sucrose metabolism (five genes), with the highest enrichment factors (Rich Factor) of 0.24 and 0.22, respectively. (**B**) Downregulated DEGs showed the highest enrichment in ABC transporters (27 genes), followed by citrate cycle (TCA cycle) and fatty acid degradation. These results indicated that *ppc* deletion disrupts central carbon metabolism and transmembrane transport processes.

**Fig 6:**
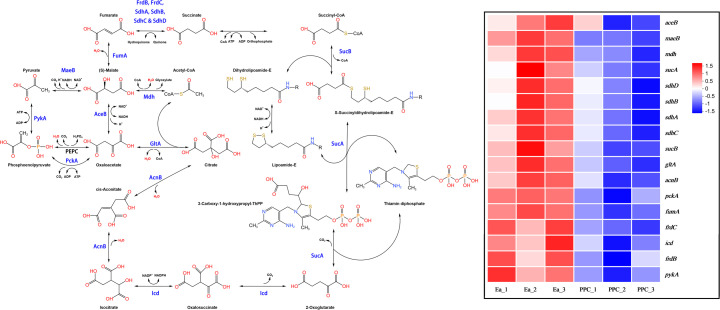
Altered metabolic pathways in EaΔppc revealed by KEGG analysis. Key genes involved in the tricarboxylic acid (TCA) cycle and pyruvate metabolism were downregulated in the mutant. The heat map showed the differential expression level among these genes.

KEGG analysis was used to explore the pathways of DEGs, which showed that genes with significant upregulation and downregulation were mainly associated with the category metabolism, accounting for 58.2% (135/232) of all pathways. In addition, DEGs were mainly concentrated in ABC transporters (27). As a large family of membrane proteins, ABC transporters participate in various important cellular processes and can realize the transmembrane transport of various substances, including the import of nutrients such as amino acids and carbohydrates, playing an important physiological role in promoting cell growth (30). According to the KEGG enrichment analysis, the significantly upregulated genes were mainly concentrated in purine metabolism (6), followed by starch and sucrose metabolism (5) (Fig. 5B). The gene enrichment in these two pathways was also the highest. By contrast, genes related to the tricarboxylic acid cycle (TCA) and fatty acid degradation had the highest enrichment among downregulated genes (Fig. 5B). These results indicated that the deletion of the *ppc* gene successfully downregulated the TCA cycle, as well as the related pyruvate metabolism to a certain extent, and affected the metabolism of various cellular nutrients (Fig. 6).

### Analysis of the polysaccharide synthesis pathways

**Fig 7:**
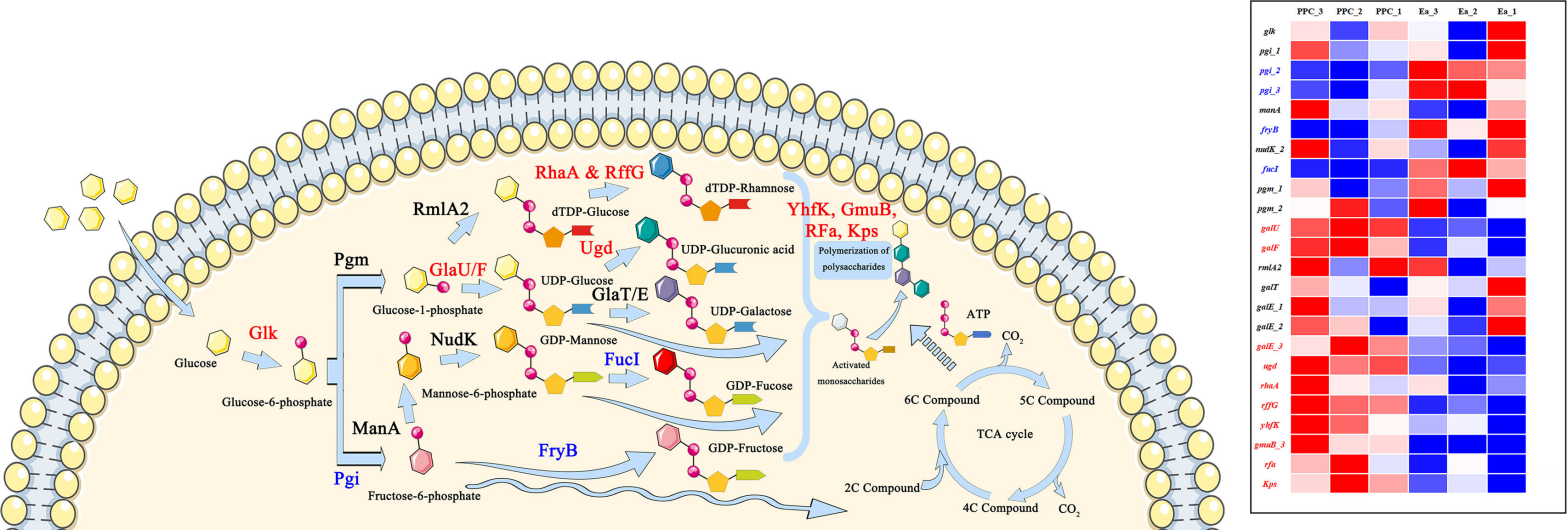
Main synthesis network and key enzymes of microbial polysaccharides. The mutant strain exhibited enhanced biosynthesis of mannose-rich polysaccharides through coordinated changes in key enzymes.

Microbial exopolysaccharide synthesis has certain common features, involving many common pathways and key enzymes (Fig. 7). In this study, based on the comparison and annotation of the transcripts of the original strain, it was found that some genes showing up- or downregulated expression were involved in microbial exopolysaccharide synthesis in the mutant strain (Table 5).

**TABLE 5:** Effective genes of transcriptional upregulation or downregulation in polysaccharide anabolism of mutant strain

Gene ID	Gene name	Description
EAChromosome_1_04283	*glk*	Glucokinase
EAChromosome_1_00741	*pgi_1*	Glucose-6-phosphate isomerase
EAChromosome_1_01198	*pgi_2*	Glucose-6-phosphate isomerase
EAChromosome_1_01199	*pgi_3*	Glucose-6-phosphate isomerase
EAChromosome_1_03233	*manA*	Mannose-6-phosphate isomerase
EAChromosome_1_02276	*fryB*	Fructose-like phosphotransferase enzyme IIB component 1
EAChromosome_1_04330	*nudK_2*	GDP-mannose pyrophosphatase NudK
EAChromosome_1_04682	*fucI*	L-fucose isomerase
EAChromosome_1_01947	*pgm_1*	Phosphoglucomutase
EAChromosome_1_02674	*pgm_2*	Phosphoglucomutase
EAChromosome_1_03566	*galU*	UTP—glucose-1-phosphate uridylyltransferase
EAChromosome_1_04006	*galF*	UTP—glucose-1-phosphate uridylyltransferase
EAChromosome_1_00613	*rmlA2*	Glucose-1-phosphate thymidylyltransferase 2
EAChromosome_1_01997	*galT*	Galactose-1-phosphate uridylyltransferase
EAChromosome_1_01998	*gal_E1*	UDP-glucose 4-epimerase
EAChromosome_1_02591	*gal_E2*	UDP-glucose 4-epimerase
EAChromosome_1_03975	*gal_E3*	UDP-glucose 4-epimerase
EAChromosome_1_03987	*ugd*	UDP-glucose 6-dehydrogenase
EAChromosome_1_00541	*rhaA*	L-rhamnose isomerase
EAChromosome_1_00612	*rffG*	dTDP-glucose 4,6-dehydratase 2
EAChromosome_1_02618	*yhfK*	Putative sugar epimerase YhfK
EAChromosome_1_04230	*gmuB_3*	Oligo-beta-mannoside-specific phosphotransferase enzyme IIB component
EAChromosome_1_02457	*rfa*	Lipopolysaccharide core biosynthesis protein
EAChromosome_1_04002	*kps*	Polysaccharide biosynthesis/export protein

Glucose taken up by the cell is first phosphorylated to generate glucose-6-phosphate by glucokinase (Glk), and the expression levels of this gene were similar in both strains. Glucose-6-phosphate can then be differentially isomerized by glucose-6-phosphate isomerase (Pgi) to form fructose-6-phosphate. The latter is further differentially isomerized by mannose-6-phosphate isomerase (ManA) to form mannose-6-phosphate. Both fructose-6-phosphate and mannose-6-phosphate enter the subsequent pyrophosphorylation reaction to produce GDP-fructose (fructose-like phosphotransferase enzyme IIB component 1, FryB) and GDP-mannose (GDP-mannose pyrophosphatase, NudK), respectively. In addition, GDP-mannose can be further differentially isomerized by L-fucose isomerase (FucI) to produce GDP-fucose. All three sugar nucleotides can be used for subsequent polysaccharide polymerization. Although there was no significant enrichment of genes associated with mannose metabolism, the fructose-related genes *pgi_2*, *pgi_3,* and *fryB* all showed varying degrees of downregulation, suggesting a higher supply of GDP-mannose for polysaccharide synthesis, in line with the results of monosaccharide composition analysis.

Glucose-1-phosphate can also be generated from glucose-6-phosphate by phosphoglucomutase (Pgm), after which it can be pyrophosphorylated by UTP-glucose-1-phosphate uridylyltransferase (GalU/GalF) to generate UDP-glucose. UDP-glucose undergoes differential isomerization by either galactose-1-phosphate uridylyltransferase (GalT) or UDP-glucose 4-epimerase (GalE) to produce UDP-galactose. Additionally, UDP-glucose can be oxidized and dehydrogenated by UDP-glucose 6-dehydrogenase (Ugd) to produce UDP-glucuronic acid. All three of these sugar nucleotides can also participate in polysaccharide polymerization. High expression of *galU* and *galF* enhances the pyrophosphorylation of glucose-1-phosphate, promoting subsequent metabolism. Among the *galT* and three *galE* genes, only *galE_3* showed significant upregulation, which also contributed to the utilization of UDP-galactose in polysaccharide polymerization. Moreover, high expression of the *udg* gene facilitated the participation of UDP-glucuronic acid in this process.

Interestingly, glucose-1-phosphate can also be pyrophosphorylated by glucose-1-phosphate thymidylyltransferase 2 (RmlA2) to generate dTDP-glucose, which can then be isomerized by either L-rhamnose isomerase (RhaA) or dTDP-glucose 4,6-dehydratase 2 (RffG) to generate dTDP-rhamnose. The expression of *rmlA2* was not significantly upregulated and did not affect the subsequent metabolism of rhamnose. By contrast, both *rhaA* and *rffG* showed very high expression levels, indicating the production of a large amount of dTDP-rhamnose. This deoxyribonucleotide sugar could be utilized for polysaccharide synthesis, but this is contrary to the results of monosaccharide composition.

At the same time, genes encoding putative sugar epimerase YhfK, oligo-beta-mannoside-specific phosphotransferase enzyme IIB component, lipopolysaccharide core biosynthesis protein, and polysaccharide biosynthesis/export protein, which are involved in polymerization of polysaccharides, were significantly upregulated in the mutant compared with wild-type Ea.

The differential expression of these genes may be an important reason why the mutant strain exhibited significantly improved utilization of carbon sources and produced exopolysaccharides in fermentation.

### Analysis of the phosphotransferase system

In addition, the analysis of DEG transcription levels also showed that several components of the phosphotransferase system (PTS) and mannose permease were upregulated or downregulated to varying degrees (Fig. S1). Widely distributed in bacteria, the phosphotransferase system transfers free energy through phosphorylation to drive the uptake of carbohydrates through the cell membrane, serving as the bacterial carbohydrate transport system (31). It also has powerful regulatory capabilities that influence the central carbon and nitrogen metabolism (32, 33).

PTS cytoplasmic phosphotransferase I (EI) is encoded by the *ptsI* gene and plays multiple roles in PTS (34), including phosphate group transfer, systemic regulation, nonspecific effects, as well as energy conversion and storage (35). In *Escherichia coli*, EI serves as the terminal phosphotransferase receiving phosphate groups from phosphoenolpyruvate (PEP) and transferring them to downstream histidine phosphotransfer proteins. This process is the first step of the PTS phosphorylation cascade and provides the necessary energy for subsequent phosphorylation and transport of sugars while promoting gluconeogenesis (36). In the differentially expressed genes of EaΔppc, all three segments of the *ptsI* gene exhibited downregulation, which was consistent with the trend of the phosphoenolpyruvate carboxylase gene *ppc* and the phosphoenolpyruvate carboxykinase gene *pckA*. Mannose permease is an important component of the EII complex in the PTS, which is the main glucose transport system in bacteria and couples carbohydrate transport with phosphorylation (37). Since the differential expression trends of the structural domain component genes were different, it is hypothesized that gene knockout affected the ability to transport and phosphorylate carbohydrates, resulting in the accumulation of exopolysaccharides.

### Proposed regulatory links between *ppc* deletion and global metabolism

The deletion of the *ppc* gene may not only interrupt the conversion of phosphoenolpyruvate (PEP) to oxaloacetate but also cause a broader disturbance in the metabolic network of *E. aerogenes*. Loss of phosphoenolpyruvate carboxylase (PEPC) activity decreases the anaplerotic supply of oxaloacetate to the tricarboxylic acid (TCA) cycle (Fig. 6), thereby reducing flux through central carbon metabolism. As a consequence, PEP and pyruvate may accumulate and be redirected toward alternative routes, including carbohydrate transport and exopolysaccharide (EPS) biosynthesis (Fig. 7). In parallel, altered intracellular PEP availability can modulate the phosphotransferase system (PTS)—which couples carbohydrate uptake with phosphorylation—thus affecting sugar transport efficiency and cellular energy balance (Fig. S1). These combined effects are likely to trigger compensatory transcriptional responses to restore metabolic homeostasis, providing a plausible explanation for the extensive differential gene expression observed in EaΔppc. A schematic model summarizing these potential regulatory connections is provided to illustrate the proposed relationships between *ppc* deletion, carbon-flux redistribution, and mannose-rich EPS overproduction. Collectively, these connections should be viewed as a hypothesis-generating model derived from transcriptome-level correlations and will require targeted experiments to establish causality.

From a translational perspective, the mannose-rich EPS reported here could be valorized via downstream depolymerization and recovery. Conceptually, EPS depolymerization could be achieved by controlled chemical hydrolysis or enzyme-assisted processing, followed by fractionation and purification to recover mannose-enriched EPS-derived products (38, 39). While optimization and experimental validation of these downstream steps are beyond the scope of the present study, these pathways could provide testable directions for future work aimed at valorizing the EPS phenotype.

### Conclusions

In this study, the *ppc* gene encoding phosphoenolpyruvate carboxylase was successfully knocked out from the genome of *E. aerogenes* IAM1183 (Ea) using triparental conjugation, and the resulting strain EaΔppc showed obvious viscosity enhancement compared with the original strain both on plates and in shake flasks. Transcriptomic analysis showed that 149 genes were significantly upregulated and 332 downregulated in the glycogenic mutant. The genes with significant changes were mainly related to metabolism, accounting for 58.2% of the pathways with significant differences. Pathway enrichment analysis also showed that the DEGs were mainly related to purine, carbohydrate, and lipid metabolism, the TCA cycle, as well as membrane transport, indicating that the *ppc* gene knockout successfully downregulated the tricarboxylic acid cycle and affected the metabolism of a variety of cellular nutrients. It was speculated that *ppc* knockout enhanced the pathway of exopolysaccharide synthesis in microorganisms but also affected the transport and phosphorylation of carbohydrates in cells, which may contribute to a special phenotype of exopolysaccharide production.

Until now, only a limited number of studies have focused on the application of *E. aerogenes* in carbohydrate production. This study reported a novel *E. aerogenes* mutant with a mannose-rich exopolysaccharide phenotype induced by phosphoenolpyruvate carboxylase inactivation, although free mannose was not isolated and purified from extracellular polysaccharide (EPS), the high mannose content and considerable EPS yield make EaΔppc a potential candidate strain for subsequent extraction of mannose. Overall, this work provides a new method for producing mannose-rich EPS in *E. aerogenes*, as well as broadening our knowledge of the metabolic network of this highly promising non-model organism.

## Data Availability

The raw sequencing data have been uploaded to the NCBI’s Sequence Read Archive (SRA) database under the following accession numbers: *Enterobacter aerogenes* IAM1183, SRR33234680, SRR33234681, and SRR33234682; EaΔppc, SRR33234677, SRR33234678, and SRR33234679.

## SUPPLEMENTAL MATERIAL

Fig. S1 aem.01895-25-s0001.docx Several components of the PTS presented varying degrees of differential expression.

ASM does not own the copyrights to Supplemental Material that may be linked to, or accessed through, an article. The authors have granted ASM a non-exclusive, world-wide license to publish the Supplemental Material files. Please contact the corresponding author directly for reuse.
