# Supplementary material for: Transcriptome profiling of a novel Enterobacter aerogenes mutant with mannose-rich exopolysaccharide phenotype induced by phosphoenolpyruvate carboxylase inactivation
Source: Appl Environ Microbiol. 2026 Mar 19;92(4):e01895-25. doi: 10.1128/aem.01895-25 (PMC13101499; doi:10.1128/aem.01895-25)
Supplement: Fig. S1 — Several components of the PTS presented varying degrees of differential expression. [file aem.01895-25-s0001.docx]

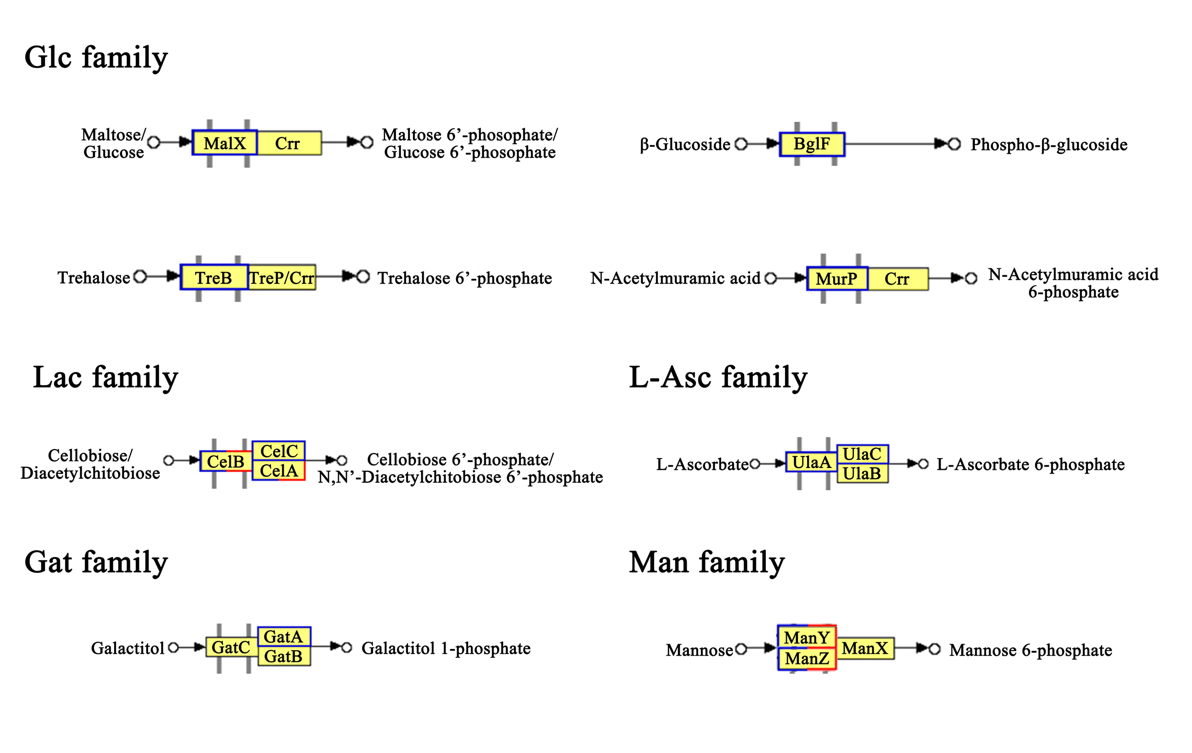


**Figure S1 Several components of the phosphotransferase system (PTS) presented varying degrees of differential expression.** Schematic representation of the Glc, Lac, Gat, L-Asc and Man PTS families, showing their respective saccharide substrates and phosphorylated products. Yellow boxes denote annotated PTS permease and EII components in *E. aerogenes*. Genes outlined in blue are significantly down-regulated, whereas those outlined in red are significantly up-regulated in EaΔppc compared with Ea based on transcriptome analysis.
